# Shorter spontaneous fixation durations in infants with later emerging autism

**DOI:** 10.1038/srep08284

**Published:** 2015-02-06

**Authors:** Sam V. Wass, Emily J. H. Jones, Teodora Gliga, Tim J. Smith, Tony Charman, Mark H. Johnson, BASIS team, Simon Baron-Cohen, Rachael Bedford, Patrick Bolton, Susie Chandler, Kim Davies, Janice Fernandes, Holly Garwood, Kristelle Hudry, Helen Maris, Greg Pasco, Andrew Pickles, Helena Ribiero, Leslie Tucker, Agnes Volein

**Affiliations:** 1 Medical Research Council Cognition and Brain Sciences Unit, Cambridge, UK; 2 Centre for Brain and Cognitive Development, Birkbeck, University of London, UK; 3 King's College London, Institute of Psychiatry, Psychology & Neuroscience, Department of Psychology, UK; 4 Autism Research Centre, University of Cambridge, Cambridge, UK; 5 Institute of Psychiatry, King's College, University of London, London, UK; 6 King's College, University of London, London, UK; 7 Centre for Brain and Cognitive Development, Birkbeck College, University of London, UK; 8 Central European Univerity, Budapest, Hungary

## Abstract

Little is known about how spontaneous attentional deployment differs on a millisecond-level scale in the early development of autism spectrum disorders (ASD). We measured fine-grained eye movement patterns in 6-to 9-month-old infants at high or low familial risk (HR/LR) of ASD while they viewed static images. We observed shorter fixation durations (i.e. the time interval between saccades) in HR than LR infants. Preliminary analyses indicate that these results were replicated in a second cohort of infants. Fixation durations were shortest in those infants who went on to receive an ASD diagnosis at 36 months. While these findings demonstrate early-developing atypicality in fine-grained measures of attentional deployment early in the etiology of ASD, the specificity of these effects to ASD remains to be determined.

In developmental psychopathology it is increasingly recognised that we should move away from ‘static neuropsychological deficit’ models of disease towards dynamic, developmentally plausible nosological models^1^,^2^,^3^,^4^. For example, work with individuals with genetic disorders such as Williams syndrome has suggested that early-developing abnormalities in attention orienting may impair the subsequent learning of behaviours such as gaze following, thus leading to impairments across other domains including social communication and number perception^5^,^6^,^7^. This suggests the importance of studying early attentional control as a ‘hub’ domain upon which later behaviours are built^2^,^7^,^8^. Within autism spectrum disorders (ASD), atypicalities in attention have been suggested as a key candidate mechanism in the ontogenesis of the cognitive phenotype^9^. Here, we examine the potential contribution of attention atypicalities to the emergence of symptoms of ASD by examining the very early development of spontaneous attention deployment in infants with later emerging ASD.

Much of our information intake in our highly visual species is controlled through oculomotor orienting behaviours. When viewing a visual array we make a sequence of rapid eye movements (saccades) interspersed with moments of relative stability (fixations) during which the light from objects of interest is projected onto the fovea, the most sensitive part of the retina, while visual encoding occurs^10^,^11^. The duration of each fixation (which ranges from a hundred to several thousand milliseconds) is influenced by exogenous properties such as object movement and luminance, as well as by endogenous properties such as the participant's processing speed, vigilance levels and interest in the information at the point fixated^12^,^13^.

The average duration of fixations shows high test-retest reliability of individual differences, both between individual infants^14^ and adults^15^. Of note, findings also suggest high consistency is observed between fixation durations recorded during the presentation of static stimuli and of complex, naturalistic stimuli^14^. These stable individual differences relate to aspects of later behaviour^16^, and to structural and functional differences in the adult brain^15^. However, Yu and colleagues also noted that phasic changes in fixation duration during a task can relate to learning performance: they found that infants who performed effectively on a learning task showed stable patterns of fixation durations across the familiarisation period, whereas those infants who performed less well had periods of shorter fixations^17^. In adults at least, fixation durations have also been shown to change as a function of time. For example, during the continuous viewing of a static scene, fixation duration increases as saccadic amplitude decreases; this has been interpreted in terms of a shift from ‘global’ to ‘focal’ modes of scanning^18^,^19^. Based on animal work, Aston-Jones and colleagues have suggested that this modulation of attentional states may be modulated by the the noradrenergic locus coeruleus (LC) arousal system^20^.

Such spontaneous eye movement patterns appear to differ in children and adults with ASD. For example, Kemner and colleagues^21^ found that 8-12-year-old children with ASD made more saccades (reflecting shorter fixation durations) than children with typical development or ADHD when viewing static geometric shapes (see also Refs. 22, 23 - although findings on fixation durations in adults with ASD are not consistent^24^). They also found that whilst typically developing children made more saccades (suggesting shorter fixation durations) when viewing familiar compared to unfamiliar objects, children with ASD did not^21^. Other groups have noted reduced modulation of fixation durations in individuals with ASD by task demands^25^ and semantic content^26^,^27^,^28^. Thus, there is evidence that older individuals with ASD do not show variability in fixation duration contingent on familiarity and task demands.

Recent research into the very early development of the cognitive phenotype of ASD during infancy has identified a number of aspects of atypical behaviour that emerge around the 12-month-boundary (see ref. 29 for a review). Problems specific to disengaging attention emerge around 12-15 months^30^,^31^. Differences in joint attention (reduced likelihood of following gaze or point cues) also appear to emerge around the start of the second year, but not to be detectable earlier in development^29^. This opens the question of whether these later atypicalities arise downstream, *as a result* of abnormalities in other aspects of development. Atypical visual scanning, un-modulated by contextual information (e.g. the presence of social cues) or with fixations that are generally too short or too long, could impact on processing and learning from social interaction. Answering this question is critical to untangling the complex causal pathways associated with the development of ASD.

To our knowledge no previous research has examined how spontaneous visual attention differs at the millisecond-level scale early in the development of ASD. To address this, we measured eye movement patterns using a 50 Hz remote corneal reflection eyetracker while infants viewed static scenes, one of which was a face, while seated on their caregiver's lap. Approximately half of our participants had an older sibling with ASD and were therefore considered at ‘high risk’ (HR) of developing ASD themselves; the other half did not, and were therefore considered at ‘low risk’ (LR) of ASD. Additionally, we examined associations with ASD clinical outcome (assessed at 36 months), in order to allow for comparison between those HR infants who later received a diagnosis of ASD (HR-ASD) with those who did not (HR-no ASD).

Our primary set of analyses focuses on the median duration of fixations produced by each child. We examined risk (HR vs LR) and outcome group (HR-no ASD/HR-ASD/LR) differences in median fixation duration (collapsing across fixations directed at different components of the slide). As a secondary analysis we also examined the continuous relationship between median fixation duration and later symptoms of ASD, as operationalized by Autism Diagnostic Observation Schedule (ADOS) social-communication total scores. This provides an assessment of whether early fixational control continuously relates to later variation in social communication skills across risk groups. Such a 'dose-dependent' relation would provide further indirect support for a causal model in which fixation behaviors have a cascading effect on later socio-communicative development. Third, we examined whether the risk group differences we identified (HR vs LR) are also present in a separate cohort for which data collection is still ongoing, and for whom risk group but not outcome group status information is currently available (‘Cohort 2’). To assess stimulus-related influences on fixation duration, we then examined group differences in fixation duration to each Area of Interest on the static slide (face, phone, car, scrambled face, bird, other), in order to establish whether differences in fixation duration were attributable to a particular class of objects. Finally, we report on supplementary analyses looking at saccadic amplitude and variability in fixation duration.

## Results

### Median fixation duration

#### Risk group/outcome group analyses

Table 1 shows demographic and descriptive data for each group. Our primary analysis was to examine whether fixation durations recorded varied as a function of risk group (HR/LR) and diagnostic outcome information (HR no ASD/HR ASD/LR).

**Figure 1:**
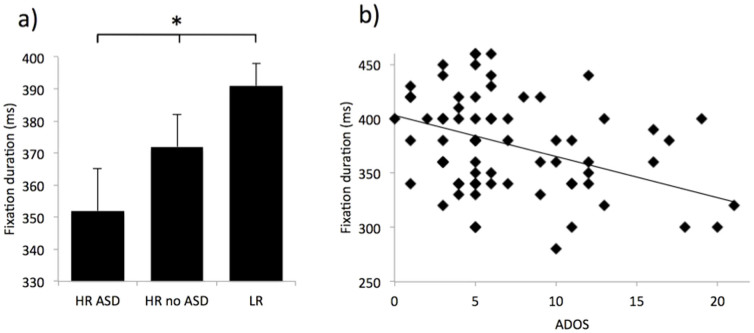
(a) Median fixation duration data by outcome group (HR-ASD N = 15, HR-no ASD N = 30, LR N = 49). Calculations are weighted as described in the text. Stars show the significance of the analyses reported in the text. Error bars show Standard Error of the Means. (b) Scatterplot showing the relationship observed across all infants between fixation duration during infancy and ADOS scores at 36 months. The linear regression line shows the significant relationship described in the text.

Figure 1 shows the average fixation duration data obtained for different individual participants. All averages were calculated using a median average as medians are less susceptible to skew (fixation durations, as in all reaction time measures, are often positively skewed) while remaining representative of the average of a distribution. Figure S1 in the Supplementary Materials (SM) (p.6) shows a histogram and a scatterplot of these per-participant averages. A Kolmogorov-Smirnov (KS) test confirmed that data were normally distributed (*D*(94) = .08, *p* = .16). All calculations presented here are weighted by the number of fixations obtained from each infant, but a non-weighted analysis was also conducted with similar results (see SM p.9). A univariate ANOVA on fixation durations weighted by number of fixations with Risk (HR, LR) as between-subjects variable revealed a significant main effect of Risk, with HR infants showing shorter fixation durations than LR infants (*F*(1,92) = 6.09, *p* = .015, η^2^ = .062). A similar ANOVA on fixation durations weighted by number of fixations with diagnostic Outcome (HR no ASD vs HR ASD vs LR) as between-subjects variable showed a significant effect of Outcome (*F*(2,91) = 3.74, *p* = .028, η^2^ = .076). Post-hoc Tukey tests showed that fixation durations were significantly shorter in the HR-ASD group than the LR group (*p* = 0.029) while the HR-no ASD group did not significantly differ from either the LR (*p* = 0.28) or HR-ASD groups (*p* = 0.48).

#### Relationship to ADOS-G social communication scores

Additionally, we examined relations with dimensional outcome phenotypes by regressing 36-month ADOS-G social communication total scores on fixation durations weighted by number of fixations. Shorter fixation durations at 8 months were associated with higher scores (worse symptoms) on the ADOS social communication total score at 36 months (*R*^2^ = 0.06; *F*(1,90) = 6.20, *p* = 0.015; *β* = −.25, p = 0.015); see Figure 1b.

#### Cohort 2

Data collection is ongoing for a subsequent cohort of infants, who participated in a similar paradigm. For this cohort, risk group status but not outcome group status is currently available. Analyses of these data are reported in the SM (pp. 3-6). In short, we found in a separate sample of 94 infants (HR N = 70; LR N = 24) that risk group differences replicate in this cohort, with shorter average fixation durations observed in the HR relative to the LR infants (p = 0.037).

### Fixation durations by Area of Interest

Here we examined the possibility that risk group differences might be attributable to differences in the durations of fixations to one but not to other categories of visual stimulus. In order to assess this we calculated independently the durations of fixations for each object being fixated at the time (Table 2). Fixations obtained for the separate non-face AOIs (car, phone, scrambled face, phone) were pooled together into a separate ‘non-face’ category. We first examined effects of risk group. A repeated-measures ANOVA on fixation durations by stimulus (face vs non-face vs empty) and risk (HR, LR), indicated a significant effect of stimulus (F(2,153) = 109.03, p <0.001, η^2^ = .59; Face>non-face>empty), but no main effect of risk (F(1,154) = .37, p = 0.54, η^2^ = .002), and no stimulus by risk interaction (F(2,308) = .24, p = 0.68, η^2^ = .002). We then looked at the effects of outcome group. A similar repeated-measures ANOVA on fixation durations by stimulus (face vs non-face vs empty) and outcome (HR-ASD, HR-no ASD, LR), indicated a significant effect of stimulus (F(2,62) = 57.23, p <0.001, η^2^ = .65; Face>non-face>empty), a main effect of outcome (F(2,63) = 3.36, p = 0.041, η^2^ = .096; ASD<HR-noASD p = 0.045; ASD< LR p = 0.06), but no stimulus by outcome interaction (F(4,124) = .913, p = 0.46, η^2^ = .029). Thus, children both with and without later ASD showed longer fixations to faces than other AOIs (see Table 2), and outcome group effects on fixation duration were consistent across AOIs. See SM p.12 for equivalent analyses on our Cohort 2 dataset.

### Saccadic amplitude

We then explored whether group differences in fixation duration might be related to instability in maintaining fixation on particular targets. If this were the case, we would also see saccades of shorter amplitude in high-risk infants. However, average (S.E.M.) saccadic amplitude for the three groups was 3.4 (0.1)° for the HR-no ASD group, 3.3 (0.2)° for HR-ASD, 3.2 (0.08)° for LR. A univariate ANOVA on saccadic amplitude by Risk (HR, LR) weighted by number of usable saccades obtained revealed no significant main effect of Risk, (*F*(1,92) = 1.08, *p* = .30, η^2^ = .012) (see also Figure S2 – SM p.14). A similar ANOVA including diagnostic outcome information (HR no ASD vs HR ASD vs LR) showed no significant effect of Outcome (*F*(2,91) = 0.02, *p* = .90 η^2^ = .00). Thus, groups did not differ in terms of fixation stability; differences in fixation duration can thus be attributed to more frequent shifts between regions in the HR-ASD group.

### Variability in fixation duration

To further explore our observation of shorter fixation durations we examined intra-individual variability in the duration of individual fixations. First, we examined the distributional properties of fixation durations obtained (see SM p.14). In brief, our analyses suggested that group differences were largely driven by differences in the tau component of the distribution, approximating to the skewedness, or tail. LR infants returned more positively skewed distributions of fixations.

Second, we examined average intra-participant variance in fixation duration; this was higher in the LR group (see Figure S3, p.15). A number of calculations were conducted to assess whether these differences reached significance (SM pp. 15–16). In sum, the risk group difference was found to be significant (p = .007, η^2^ = .039); a significant effect of Outcome (*p* = 0.029, η^2^ = .075) was also observed, but posthoc tests indicated that the HR-no ASD group did not differ significantly from the HR ASD group (p = 0.99; see SM p.14). These analyses suggest that intra-individual variance in fixation duration was higher in the LR infants in our sample.

Previous research has suggested that fixation durations can change over time during scene exploration^18^,^19^. As a third additional analysis, therefore, we also examined the modulation of fixation duration by becoming familiar with the image by examining how fixation durations change over time during the course of the trial (see Figure S4 and SM pp. 16–18). In sum, LR infants showed an increase in fixation duration during the course of the trial (p = .047, η^2^ = .008), which is consistent with the pattern documented in adults^18^. In contrast, fixation durations for HR infants did not change significantly during the course of the trial (p = .65, η^2^ = .001). However, the HR-no ASD group did not differ significantly from the HR-ASD group on this measure (p = .30, η^2^ = .041).

## Discussion

We examined the relation between fixation duration and risk status, ASD outcome and social communication abilities in young infants at high and low familial risk for ASD. Across two cohorts we observed that infants at high risk for ASD showed shorter fixation durations than infants at low risk. In the cohort for whom 36-month outcome assessments were available we also identified a significant main effect of outcome, with fixation durations shortest at 6- to 9-months in those high-risk infants who went on to have a diagnosis of ASD at 36 months; however a group comparison by ASD outcomes within the HR group was not significant. In addition, we examined the relationship with dimensional outcome phenotypes across both HR and LR groups and found that shorter fixation durations during early infancy were associated with higher levels of social communicative difficulties at 36 months. Together, our findings of risk and outcome group differences suggest for the first time that shorter fixation durations may be part of the very early manifestation of ASD. Continuous relations with ASD symptoms indicate that individual differences in early fixation behaviour are related to variation in the development of later social and communication skills, consistent with the possibility of a role for fixation behaviour in socio-communicative learning.

One caveat to this conclusion is evidence of a relation between fixation durations and concurrent performance on a more general cognitive task (the Mullen Scales of Early Learning Composite (ELC) Score). 8-month Mullen ELC scores showed a significant relationship with 8-month fixation duration. High-risk infants showed significantly lower scores on the 8-month Mullen ELC than low risk infants, although it is important to note that the mean score of 90 does not fall in the developmentally delayed range. Covarying for cognitive scores rendered risk group effects on fixation duration marginally non-significant (p = .08), and relations with continuous variation in later ASD symptoms non-significant (p = .12). Of note, however, no relationship was found between fixation duration at 8 months and Mullen Early Learning Composite scores at 36 months (the same age that the ADOS-G was administered). Further, in a regression analysis with 8-month fixation duration as the dependent variable and 8-month Mullen, 36-month Mullen and 36-month ADOS as the predictors, 36-month ADOS was retained as the strongest predictor. Thus, crucially, fixation durations are not related to cognitive level at 36 months, but are related to ASD symptoms at 36 months. Nevertheless, future work should investigate the degree to which the present results are specific to ASD, or would be seen in other groups with compromised development. This will require future work with prospectively assessed population samples and other groups at risk for neuro-developmental delays, such as infants born pre-term.

One further limitation of the present analyses is that viewing data were recorded only during the presentation of static scenes, rather than the more complex, dynamic scenes encountered in real-world settings (cf. ref. 43). Although previous work has suggested that inter-individual differences in fixation duration are stable across different types of viewing material, in infants^14^ and in adults^15^, our recent findings also suggest that different components of attentional control may influence fixation durations across static and dynamic scenes^14^. Future work should investigate this issue in more detail. A final limitation of the present findings is that fixation durations were assessed based on eye-tracker data, rather than on hand coding of fixation durations (cf refs. 38, 39) or EOG^21^. In the SM we present a number of analyses conducted to evaluate this, confirming for example that group differences were largely independent of data quality.

Despite these caveats, our findings may offer some important indications as to etiological mechanisms within the autistic behavioural phenotype. Research suggests that many aspects of abnormal behaviour in ASD emerge beyond 6 months of age, and are not detectable at the earlier ages studied here^29^,^30^,^34^. Future work should examine longitudinal change in the fixation duration, and their relation to the emergence of disengagement difficulties observed in 12- to 14-month-old infants with later ASD in experimental^30^,^31^ and naturalistic^31^ contexts.

Key to understanding developmental changes will be to understand the causes of the shorter fixation durations we observed in the present study. Here, the secondary analyses presented in the Results are informative. First we examined fixation durations by Area of Interest and concluded that group differences appear consistent across all the types of visual stimulus presented – including faces and non-faces. This suggests that fixation duration atypicalities are not specific to social stimuli, like other previous measures (cf. e.g. ref. 35) – and may reflect general visual information processing or attentional differences. Next we examined saccadic amplitude, and identified no group differences. This suggests that the oculomotor control atypicalities identified in some adults with ASD are not the cause of the shorter fixation durations observed^36^,^37^.

Perhaps most revealing are the differences reported in the final section of the results, where we looked at variability in fixation duration. Pannasch and colleagues^18^ reported that during the viewing of a novel scene, typical adults show an initial ‘scanning’ phase, characterised by shorter fixation durations, followed by a subsequent phase of longer fixation durations. We found this pattern of change in fixation duration over time in our LR sample, which is the first demonstration of this behavior during infancy (Figure S4). In contrast, fixation durations in our HR sample were less variable across the trial, suggesting decreased flexibility in orienting behaviors. Outcome group analyses were inconclusive (see SM pp. 14-19). This may be due to lack of power; further work should assess whether these effects replicate in other cohorts before strong conclusions can be drawn.

Our findings of reduced flexibility in millisecond-level response behaviours in the broader phenotype of ASD may link to the literature on how orienting behaviors are influenced by the noradrenergic locus coeruleus (LC) arousal system^20^. Based on animal work, Aston-Jones and colleagues have suggested that the LC may play a role in modulating attentional states, and shifting between ‘focused’ and ‘scanning’ attentional states; at higher levels of arousal, they suggest, the ability to shift between these different modes can be impaired, leading to a predominance of ‘scanning’ over ‘focused’ attentional states^20^,^38^. Working with human infants, Bronson^38^ found in typically developing 13-week-olds that higher levels of arousal (measured via differentials of pupil diameter and physical activity levels) was associated with fewer long fixations when looking at static geometric stimuli (see also Refs. 14, 38, 39,14, 38, 39,14, 38, 39). Wass and Smith found that increased arousal (assessed via tonic pupil size) was associated with shorter fixation durations during static scene viewing in typical 11-month-old infants^14^. Higher arousal in ASD and related conditions at later stages of development has been reported using a variety of indices, including tonic pupil size^40^, respiratory sinus arrhythmia, baseline heart rate^41^ and galvanic skin response^42^ – but has not to our knowledge been researched at the very early stages of development studied here.

## Methods

### Participants

Experimental protocols were approved by the Birkbeck College Research Ethics Committee, and all methods were carried out in accordance with the approved guidelines. Informed consent was obtained from all subjects.

Participants in Cohort 1 were 54 infants at high (HR) and 50 infants at low familial risk (LR) for ASD; infants attended lab-based testing between 6-10 months. At the time of enrolment, none of the infants had been diagnosed with any medical or developmental condition. HR infants (N = 54, 9 female) all had an older sibling (hereafter, proband) with a community clinical diagnosis of ASD. Proband diagnosis was confirmed by two expert clinicians (PB, TC) based on information using the Development and Wellbeing Assessment (DAWBA; ref. 44) and the parent-report Social Communication Questionnaire (SCQ). See Supplementary Materials (SM) p.2 for more details. Infants in the low-risk group were recruited from a volunteer database at the Centre for Brain and Cognitive Development, Birkbeck. All low-risk infants had at least one older sibling with typical development (22 female), and no first-degree relatives with ASD. None of the older siblings scored above instrument cut-off on the SCQ for ASD (≥15).

#### Outcome characterisation at 36 months

Of the 54 HR infants recruited, 53 were retained to 36 months of age when a comprehensive diagnostic assessment was undertaken. Parents completed the Autism Diagnostic Interview – Revised (ADI-R; ref. 45) and the Social Communication Questionnaire (SCQ) and toddlers were assessed with the Autism Diagnostic Observation Schedule-Generic^46^, with standard algorithms computed. Assessments were conducted by or under the close supervision of clinical researchers (i.e., psychologists, speech therapists) with demonstrated research-level reliability. In determining diagnostic outcome status, clinicians who were not blind to risk status reviewed information across 24 month and 36 month (including ADOS-G and ADI-R) visits and reached consensus according to ICD-10 criteria for ASD. The ICD-10 ASD category includes childhood autism, atypical autism, and other pervasive developmental disorders (PDD) (World Health Organisation 1993). Given the young age of the children, and in line with DSM-5, no attempt was made to assign specific sub-categories of PDD/ASD diagnosis. Seventeen toddlers met ICD-10 criteria for ASD (hereafter, HR-ASD). The remaining 36 HR toddlers did not meet criteria for ASD (hereafter HR-no ASD). Cognitive and developmental characteristics of the sample can be found in Table 1.

#### Cohort 2

Equivalent descriptions of the Cohort 2 participants are given in the Supplementary Methods (Table S2 and SM pp. 2–4).

### Methods and Procedures

#### Assessment of fixation duration

Infants were seated in their mother's laps, in front of a stimulus display monitor which subtended c. 24°. Stimuli were presented as one block, in 14 trials of 15 seconds per trial, while gaze was recorded with a 50 Hz Tobii 1750 eyetracker using Tobii Studio. This device contains an automated calibration sequence that allows infants' reported position of gaze to be tracked with an accuracy claimed by the manufacturers to average 0.5° under optimal conditions, and using standard error measurement techniques (Tobii Eye Tracker User Manual (2006) - ClearView analysis software Copyright © Tobii Technology AB, although see ref. 32).

Images presented to infants each contained five objects (each subtending c.4°); these were: face, mobile phone, bird, car, scrambled face. Other performance measures derived from this task (orienting to face and proportion looking time to the face) have, for the subset of the infants included in this analysis, previously been reported elsewhere^49^. Briefly, all groups of infants were significantly more likely to look first at the face than the other images in the slide displays. Risk group differences (HR vs LR) were identified but no significant effects of outcome group on proportion looking time to the face were observed. However, previous work did not examine how the temporal dynamics of fixations differed between groups. This was the focus of the present paper.

Fixation parsing was performed using an automated procedure^33^. This fixation parsing procedure was designed in response to analyses^33^ indicating that the fixation detection algorithms traditionally supplied with eyetrackers perform poorly on the variable quality eyetracker data obtained from infants (see also Ref. 32). Briefly, the analysis procedure was as follows: 1) smoothing was performed using a bilateral filtering algorithm^47^; 2) interpolation was performed (based on the average x- and y-coordinates since the start of the fixation to cover periods of data loss up to 150 ms; 3) velocity thresholding was performed using a velocity threshold of 35°/sec; 4) artifactual fixations and saccades were identified based on the following criteria: a) fixation is a complete fixation; b) displacement since previous fixation is >0.25°; c) average velocity during previous fixation is < 12°^/sec^; d) velocity in the three samples immediately preceding the saccade is < 12°^/sec^; e) binocular disparity is not above 3.6°; the fixation identified has a minimum temporal duration of 100 ms. The rationale and validation procedures underpinning these processing steps is outlined in detail elsewhere^33^. Saccadic amplitude was defined as the Euclidean distance (expressed in degrees of visual angle) between consecutive fixations.

The number of participants and number of fixations obtained per participant is shown in Table 2. Of those participants who attended the visit and from whom it was possible to record eyetracking data (HR-noASD 32, HR-ASD 15, LR 49) we found for a subset (HR-no-ASD 2) that the data obtained was too limited or of too poor quality for usable fixation durations to be returned by our parsing algorithm (see ref. 33 and SM pp 7-8 for further discussions of this issue). One additional child in the HR group did not return for 36-month assessment, and was thus not included in the analyses for ASD outcome. Table 1 shows demographic information for the participants from whom usable fixation duration data were obtained.

## Author Contributions

S.W. conducted preliminary data processing. S.W. and E.J. conducted all analyses and wrote the manuscript. T.G. designed experimental stimuli. T.G. and T.S. advised on aspects of the analysis. T.C. and M.J. supervised the research. BASIS team members contributed to task design and performed data collection. All authors reviewed the manuscript.

## Supplementary Material

Supplementary Information Supplementary Materials

## Figures and Tables

**Table 1:** Characteristics for all participants from whom usable fixation duration data were obtained

	HR-ASD	HR-no ASD	LR
**N (female)**	15 (8)	30 (21)	49 (29)
**Chronological age (days) – 8 month visit**	237 (37)	225 (37)	234 (36)
**Mullen ELC_SS(1) – 8 month visit**	90 (9)	96 (15)	105 (12)
**Mean (S.E.M.) fixations obtained per participant – 8 month visit**	91.5 (12.1)	71.5 (11.6)	89.7 (9.0)
**Range of fixations per participant – 8 month visit**	21-177	2-165	2-213
**Duration of usable data fragments (secs) – 8 month visit**	1.55 (0.27)	1.56 (0.19)	1.85 (0.15)
**Chronological age (months) – 36 month visit**	38.1 (2.0)	38.0 (3.3)	38.7 (3.1)
**Mullen ELC_SS(1) - 36 month visit**	101 (27)	107 (18)	116 (16)
**ADOS (total score)(2) – 36 month visit**	12.2 (3.8)	6.6 (4.8)	5.6 (4.3)
**ADI-R soc(3)– 36 month visit**	6.5 (5.2)	3.8 (5.6)	-
**ADI-R comm(4) – 36 month visit**	6.8 (5.3)	3.5 (4.5)	-
**ADI-R SBRI(5) – 36 month visit**	2.9 (2.2)	1.2 (1.9)	-

^(1)^ Mullen Scales of Early Learning^48^ Early Learning Composite Standard Score;

^(2)^ Autism Diagnostic Observation Schedule—Social and Communication Algorithm Score (ADOS-G^46^);

^(3)^ Autism Diagnostic Interview—revised^45^ - Reciprocal Social Interaction Algorithm Score;

^(4)^ Autism Diagnostic Interview—revised^45^ - Communication Algorithm Score;

^(5)^ Autism Diagnostic Interview—revised^45^ - Stereotyped/Restricted Behavior Algorithm Score.

**Table 2:** proportion fixations by area of interest (top) and fixation duration by area of interest (bottom). Both top and bottom show average (S.E.M.) data. Proportion fixation was calculated as the proportion of available fixations within that area of interest, divided by the total number of fixations available across all areas of interest. Fixation duration was calculated as the median duration (in ms) of all fixations recorded wihin that particular area of interest

	LR	HR - ASD	HR – no ASD
**Proportion fixations**			
**Birds**	0.14 (0.01)	0.13 (0.02)	0.10 (0.02)
**Scrambled face**	0.16 (0.01)	0.15 (0.02)	0.13 (0.01)
**Phone**	0.11 (0.01)	0.09 (0.01)	0.13 (0.02)
**Cars**	0.18 (0.01)	0.21 (0.03)	0.20 (0.03)
**Faces**	0.30 (0.01)	0.30 (0.02)	0.30 (0.02)
**Blank areas of the screen**	0.12 (0.01)	0.12 (0.01)	0.13 (0.01)
**Fixation duration (ms)**			
**Birds**	400 (20)	350 (30)	410 (30)
**Scrambled face**	410 (20)	350 (30)	390 (30)
**Phone**	390 (20)	380 (40)	390 (30)
**Cars**	440 (20)	450 (30)	450 (30)
**Face**	590 (30)	530 (50)	600 (60)
**Blank areas of the screen**	310 (7)	310 (10)	330 (10)
