# Supplementary material for: Shorter spontaneous fixation durations in infants with later emerging autism
Source: Sci Rep. 2015 Feb 6;5:8284. doi: 10.1038/srep08284 (PMC4319149; doi:10.1038/srep08284)
Supplement: Supplementary Information — Supplementary Materials [file srep08284-s1.doc]

SUPPLEMENTARY MATERIALS FOR:

Shorter spontaneous fixation durations in infants with later emerging autism.

Sam V. Wass, Emily J.H. Jones, Teodora Gliga, Tim J. Smith, Tony Charman, Mark H. Johnson, BASIS team

Supplementary Materials

Index of supplementary materials

1) Supplementary Methods

a) Further participant characteristics – Cohort 1 p.2

b) Cohort 2 – p.3

2) Supplementary results

i) Part 1 - Core analyses p.5

a) Scatterplots p.5

b) Median fixation duration - Cohort 2 p.6

c) Data quality p.7

d) Number of usable fixations obtained per individual p.9

e) Age p.10

f) Mullen p.10

ii) Part 2 - Fixation durations by Area of Interest p.11

iii) Part 3 - Saccadic amplitude analyses p.13

iv) Part 4 - Variability in fixation duration – p.13

a) ex-Gaussian distribution fitting p.14

b) Intra-individual variance in fixation duration p.15

c) Variance in fixation duration - change in fixation duration over time p.16

3) Supplementary References – p.19

1. Supplementary methods

a) Further participant characteristics – Cohort 1

Proband diagnosis: Most probands met criteria for ASD on both the DAWBA and SCQ (n = 44). While a small number scored below threshold on the SCQ (n = 4) no exclusions were made, due to meeting threshold on the DAWBA and expert opinion. For 2 probands, data were only available for either the DAWBA (n = 1) or the SCQ (n = 1). For 4 probands, neither measure was available (aside from parent-confirmed local clinical ASD diagnosis at intake). Parent-reported family medical histories were examined for significant medical conditions in the proband or extended families members, with no exclusions made on this basis. Screening for possible ASD in the older siblings of the low-risk group was undertaken using the SCQ, with no child scoring above instrument cut-off for ASD (>15) (one missing).

b) Cohort 2

We also wished to examine whether similar effects could be observed in our current cohort of infants from whom data collection is still ongoing. Outcome data is not yet available for this cohort.

Cohort 2 consisted of 78 HR and 34 LR infants; infants attended lab-based testing between 8-11 months (see Table S2). All HR infants had an older sibling with a community clinical diagnosis of ASD; LR controls had an older sibling with typical development and no family history of ASD. Similar inclusion criteria as were applied as for Cohort 1. All high-risk infants (N=78) had an older sibling (hereafter, proband) with a community clinical diagnosis of ASD. For 76 of these probands parents had completed either the DAWBA and/or the SCQ. 60 probands met criteria on both the DAWBA and SCQ, While a small number scored below threshold on the SCQ (n = 6) no exclusions were made, due to meeting threshold on the DAWBA and expert opinion. For 10 probands, confirmation of local clinical diagnosis was only available for either the DAWBA (n = 9) or the SCQ (n = 1). For 2 probands, neither measure was available (aside from parent-confirmed local clinical ASD diagnosis at intake).

Screening for possible ASD in the older siblings of the low-risk group was undertaken using the SCQ, with no child scoring above instrument cut-off for ASD (>15) (two missing).

Table S2 shows the participant characters for the Cohort 2 sample. This table is equivalent to Table 1 for Cohort 1.

*Table S2 – participant characteristics of Cohort 2 sample.*

*
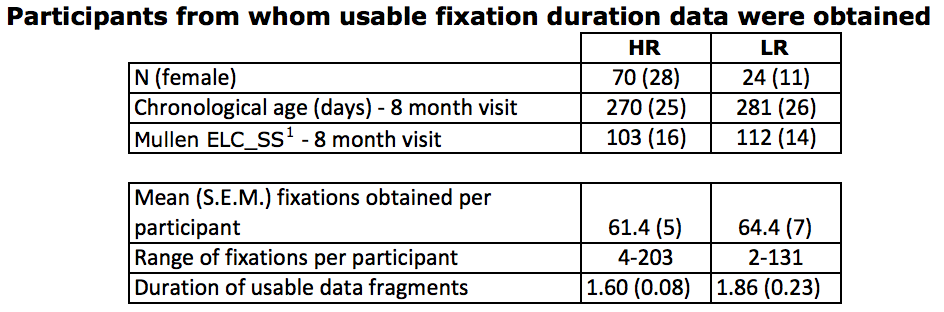
*

Methods and Procedures used for Cohort 2 were identical, with the following exceptions. First, data was collected in part on a 50Hz Tobi 1750 and in part on a 60Hz T120 eyetracker, due to technical difficulties experienced during testing. 49% of HR infants were tested on T120 and 8% of LR; split comparisons suggested group mean (of the medians) fixation durations were identical across the two eyetrackers - HR mean (standard error) was .35 (.01) secs on 1750 and .35 (.02) secs on T120. Second, 10 trials of 15 seconds per trial were presented in 3 blocks (4/3/3) interspersed with other elements of a testing battery. Third, in 3 slides the face was replaced with an item from the other four categories (see Table S2 below).

**2) Supplementary results**

*Part 1 – Core analyses*

1. *Scatterplots.* Figure S1 a) and b) shows a histogram and per-group scatterplot of the Cohort 1 data. Visual inspection of Figure S1b) reveals an outlier at c.570ms in the LR group, which did not lead to a violation of the assumption of normalcy (see K-S test reported in the main text). However, in order to ensure that this outlier was not influencing our results both analyses of group differences in fixation duration (HR vs LR and HR-ASD vs HR-no ASD vs LR) were repeated without this outlier at c.570ms in the LR group. Both results remained significant.
2. *Risk group analyses – Cohort 2.* First, the core analyses reported in Part 1 of the main text were repeated on data obtained from Cohort 2. Figure S1 c) and d) shows a histogram and per-group scatterplot of the Cohort 2 data. Figure S1d) reveals an outlier in the LR group at c.680ms. A Kolmogorov-Smirnov (KS) test confirmed that data were not normally distributed (*D*(95)=.12, *p*=.001). Therefore the analysis was conducted using non-parametric analyses. Following the techniques described in section 2d) (p.10) below, participants from whom fewer than 30 usable fixations were obtained were excluded. An Independent-Samples Mann-Whitney U test was conducted with median fixation duration as the dependent variable and Risk (HR, LR) as the independent variable. This showed a significant difference between groups (p=.037).

*Figure S1 – a) histogram showing all Cohort 1 fixation duration data that were entered into the ANOVA reported in the main text. b) scatterplot, organised by group, of the per-participant median fixation duration data from Cohort 1. Only those participants with more than 30 fixations per participant are shown. The red bars show the per-group means (of the medians), calculated as described in the main text.*

*c) histogram showing all Cohort 2 fixation duration data. d) scatterplot of the per-participant median fixation duration data from Cohort 2. The analyses conducted on these data are described above (SM p.6).*

c) *Data quality*. Our fixation parsing procedure was designed in response to analyses presented in Wass, Smith & Johnson (2012; see also Wass & Smith, 2014) suggesting that the fixation detection algorithms traditionally supplied with eyetrackers can perform poorly on the variable quality eyetracker data obtained from infants. In this paper we operationalize data quality in two ways: the first of these is flicker, the reliability of contact with the eyetracker, which we quantified as the duration of average usable data segments obtained from the eyetracker. The second is precision, the accuracy of the position of gaze reporting, which we quantified as the variance in reported position of gaze within data fragments labelled as fixations. In Wass, Smith & Johnson (2012) we present results suggesting that the results of the fixation parsing algorithms we used are (unlike the fixation parsing algorithms traditionally supplied by eyetracker manufacturers) independent of both of these parameters of data quality.

In order to verify these results, we repeated the same analyses on the present dataset. Our analyses suggested that precision did not differ significantly between groups, but that flicker (operationalized as fragment duration) was non-significantly (but consistently across phases 1 and 2) higher in HR vs LR (see Tables 2 and S2). There was no significant effect of outcome (F(2,93)=0.922, p=.40) but post-hoc tests show the HR vs LR contrast approached significance (p=.13). Regression analyses of fixation duration on fragment duration weighted by number of fixations identified significant relationships between flickeriness of eyetracker contact and fixation duration – more flickery data associated with shorter fixation durations (Cohort 1 - *r*^2^ = 0.09; *F*(1,92) = 8.86, *p* = 0.004; *β* = .30, *p* = 0.004 ; Cohort 2 - *r*^2^ = 0.2; *F*(1,93) = 22.87, *p* <0.001; *β* = .44, *p* < 0.001). This is in contrast to the identical analyses we present in Wass, Smith & Johnson (2012), where no significant relationships were identified; the reason for this may be that the relationship is stronger in the reduced quality eyetracker data common when recording from infant risk populations.

In order to assess the possibility that our findings may be influenced by these differences in data quality we conducted three separate analyses. First, we operationalized the degree of flicker as the average duration of continuous data fragments after interpolation of gaps < 150ms using the procedures outlined in Wass, Smith & Johnson (2012), and included this parameter as a covariate in our analyses (see also Wass et al., 2014). For Cohort 1, an ANCOVA on fixation durations by Risk (HR, LR) weighted by number of fixations and covarying for fragment duration revealed a significant main effect of Risk (HR vs LR), with HR infants showing shorter spontaneous fixation durations than LR infants (*F*(1,91)=4.11, *p*=.046, η^2^=.043). The same analysis for Cohort 2 also showed a main effect of Risk (*F*(1,92)=7.45, *p*=.008 η^2^=.075). A similar analysis by outcome group for Cohort 1 (HR ASD vs HR no ASD vs LR) also showed a similar pattern to results without the covariate, although effects did not reach significance (*F*(2,90)=2.35, *p*=.10 η^2^=.05). Regressing 36-month ADOS-G social communication total scores on fixation durations weighted by number of fixations and including fragment duration as a predictor revealed that fixation duration remained a significant predictor (β = -.24, *p* = 0.022).

Second, we matched a subset of the data pairwise based on data quality (following e.g. Griffith et al., 1999), according to the following procedure. For each participant in the LR group, the participant from the HR group who was most closely matched on the degree of flicker parameter of data quality (operationalized as described above) was selected. For participants in the LR group for whom a closely matched pair (defined as within 1.5 standard errors) was not available, the results of that participant were excluded. Non-matching data-points from the HR group were excluded. The comparison dataset had 49 datapoints per HR/LR group. The means (S.E.M.) of the resulting populations were: Data quality (fragment duration (secs)): HR: 1.74 (0.11), LR: 1.73 (0.11) (units are the duration of usable data segments in iterations – see Wass et al., 2012) and fixation duration (ms): HR: 340 *(8)*, LR: 380 *(8)*. Univariate ANOVAs on the mean fixation duration for each infant by group (HR, LR) revealed that fixation durations were significantly shorter in the HR than the LR infants: (*F*(1,89)=9.8, *p*=.002).

Third, we used a C++ program (GraFix; Rodriguez Saez de Urabain, Johnson & Smith, 2014) to hand-code fixation durations from a subset of 12 HR and 12 LR infants. Selection was random but stratified by data quality within each group. Comparison showed that the HR group showed shorter fixation durations than the LR group, with a similar magnitude of group differences as in the full cohort (HR M = 426 (12); LR M = 440 (12)).

We also conducted a number of further analyses to examine the possibility that other factors might influence our findings:

d) *Number of usable fixations obtained per individual*. In the main text our analyses were weighted by the number of usable fixations obtained from each individual, due to the large variability in the number of usable fixations obtained. In order to examine the possibility that any observed group differences are attributable to this weighting we also conducted an unweighted analysis excluding those individuals from whom fewer than 30 usable fixations were obtained. The Ns for this analysis were: Cohort 1: HR-ASD - 12; HR-no ASD 23; LR 38; Cohort 2: HR - 61 LR - 19. For Cohort 1, a univariate ANOVA on fixation durations by Risk (HR, LR) revealed a significant main effect of Risk (HR vs LR), with HR infants showing shorter spontaneous fixation durations than LR infants (*F*(1,71)=6.98, *p*=.01, η^2^=.089). The same analysis for Cohort 2 also showed a main effect of Risk (*F*(1,79)=13.06, *p*=.001; η^2^=.14). A similar analysis by outcome group for Cohort 1 (HR ASD vs HR no ASD vs LR) showed a significant effect of Outcome (*F*(2,70)=4.16, *p*=.02). Posthoc tests indicated that the ASD and LR groups were significantly different (*p* = 0.022); with the HR-no ASD intermediate (HR-no ASD vs HR-ASD: *p* = 0.49*,* HR-no ASD vs LR *p* = 0.23*).* Regressing 36-month ADOS-G social communication total scores on fixation durations revealed that fixation duration remained a significant predictor (*r^2^* = 0.09; *F*(1,70) = 7.15; *β* = -.31, p = 0.009).

e) *Age*. We investigated whether the group differences we observed were attributable to age by adding the infants’ age in days on the date of testing as a covariate to key analyses. For Cohort 1, an ANCOVA on fixation durations by Risk (HR, LR) covarying for age revealed a significant main effect of Risk (HR vs LR), with HR infants showing shorter spontaneous fixation durations than LR infants (*F*(1,91)=5.78, *p*=.018, η^2^=.06). The same analysis for Cohort 2 also showed a main effect of Risk (*F*(1,92)=12.37, *p*=.001; η^2^=.12). A similar analysis by Outcome (HR ASD vs HR no ASD vs LR) for Cohort 1 showed a significant effect of Outcome (*F*(2,90)=3.69, *p*=.03; η^2^=.08) Follow-up ANOVAs indicated that the ASD and LR groups were significantly different (*F*(1,61) = 6.25, *p* = 0.15); with the HR-no ASD intermediate (HR-no ASD vs HR-ASD: *F*(1,42) = 2.1, *p* = 0.14; HR-no ASD vs LR *F*(1,76) = 2.01, *p* = 0.16*).* Regressing 36-month ADOS-G social communication total scores on fixation durations with age as a predictor revealed that fixation duration remained a significant predictor (β = -.26, *p* = 0.013).

f) *Mullen*. A univariate ANOVA on Mullen Early Learning Composite (ELC) Scores at the 8-month (henceforth ‘8m Mullen’) assessment indicated that the outcome groups significantly differed in their early cognitive skills (F(2,101) = 9.36, p < 0.001); posthoc tests indicated that this was due to significantly better performance in the LR group relative to the two HR groups (LR vs HR-noASD, p = 0.002; LR vs HR-ASD, p = 0.002; HR-noASD vs HR-ASD, p = 0.71). Furthermore, fixation durations were found to relate significantly to performance on the 8m Mullen (F(1,93) = 5.52, p = 0.02). We therefore wished to investigate whether the group differences we observed were attributable to general developmental level by adding 8m Mullen scores as a covariate to key analyses. For Cohort 1, an ANCOVA on fixation durations by Risk (HR, LR) covarying for Composite Standard Scores revealed a marginally reduced effect size relative to the same analysis without 8m Mullen as a covariate. The main effect of Risk was now above an alpha level of .05, although effect sizes were comparable (*F*(1,90)=3.13. *p* = 0.08, η^2^=.08). A similar analysis by Outcome (HR ASD vs HR no ASD vs LR) for Cohort 1 again showed a comparable effect size but a non-significant effect (*F*(2,89)=2.17, *p*=.12). The same analysis for Cohort 2 showed that the main effect of Risk was significant, even with the addition of 8m Mullen (*F*(1,92)=13.08, *p*<0.001; η^2^=.13).

These findings are consistent with the hypothesis that fixation durations show a weak relationship with concurrent (8m) developmental level, which in turn shows a weak relationship with subsequent (36m) ASD diagnosis. In order to estimate the degree to which our findings relate to subsequent ASD symptoms, as opposed to more general cognitive impairment, we additionally examined the relationship of fixation duration at 8 months to Mullen ELC scores at 36 months (henceforth 36m Mullen). In contrast with the significant relationship reported in the main text, between 8m fixation duration and 36m ADOS scores, 8m fixation durations show no relation to 36m Mullen (F(1,90) = .84, p = 0.36). We conducted a backwards stepwise regression analysis with fixation durations as a dependent variable (excluding those participants from whom fewer than 30 usable fixations were obtained, as with other comparable analyses), and 8m Mullen, 36m Mullen and 36m ADOS as predictors. 36m ADOS was retained as the strongest predictor in the model (*r^2^* = 0.07; *F*(1,67) = 5.32; *β* = -.27, p = 0.024). Both 8m Mullen (*β* = .11, p = 0.36) and 36m Mullen (*β* = .008, p = 0.95) were excluded in the best-fitting model. Thus, it appears that, while fixation durations are clearly related to concurrent developmental level, they are not related to cognitive level at 36 months but they are related to autism symptoms at 36 months.

*Part 2 – Fixation durations by Area of Interest*

We performed the same calculations to examine fixation durations by area of interest for Cohort 2. The results of this analysis, which replicates that reported in Table 2 of the main text, are shown in Table S2.

*Table S2. Cohort 2 - Proportion fixations by area of interest and fixation duration by area of interest.*


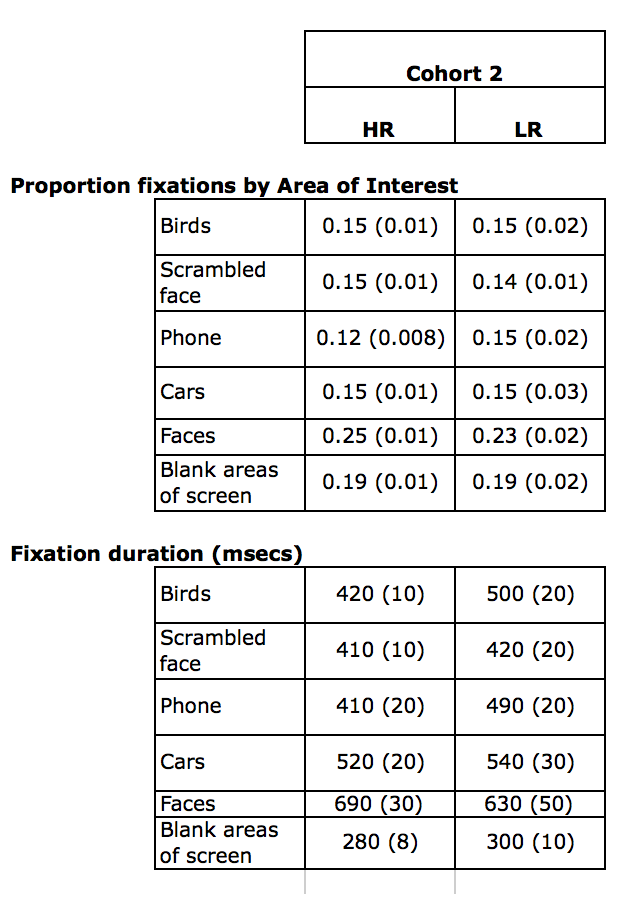


Risk effects (HR/LR) on mean fixation duration were analysed for each AOI. A repeated-measures ANOVA on fixation durations by Stimulus (face vs non-face vs empty) and Risk Group (HR, LR), crucially indicated no Stimulus by Group interaction (F(4,88) = 1.73, p = 0.19, η^2^=.019). This suggests that the general pattern observed was similar across AOIs, which is consistent with the relationship observed in Cohort 1. Thus, this does not provide evidence of modulation of group effects on fixation duration by object fixated.

*Part 3 – Saccadic amplitude analyses*

We also wished to assess whether average saccadic amplitude varies by risk and outcome group. Therefore we calculated the amplidute of all usable saccades obtained within our sample. ANOVA results are reported in the main text.


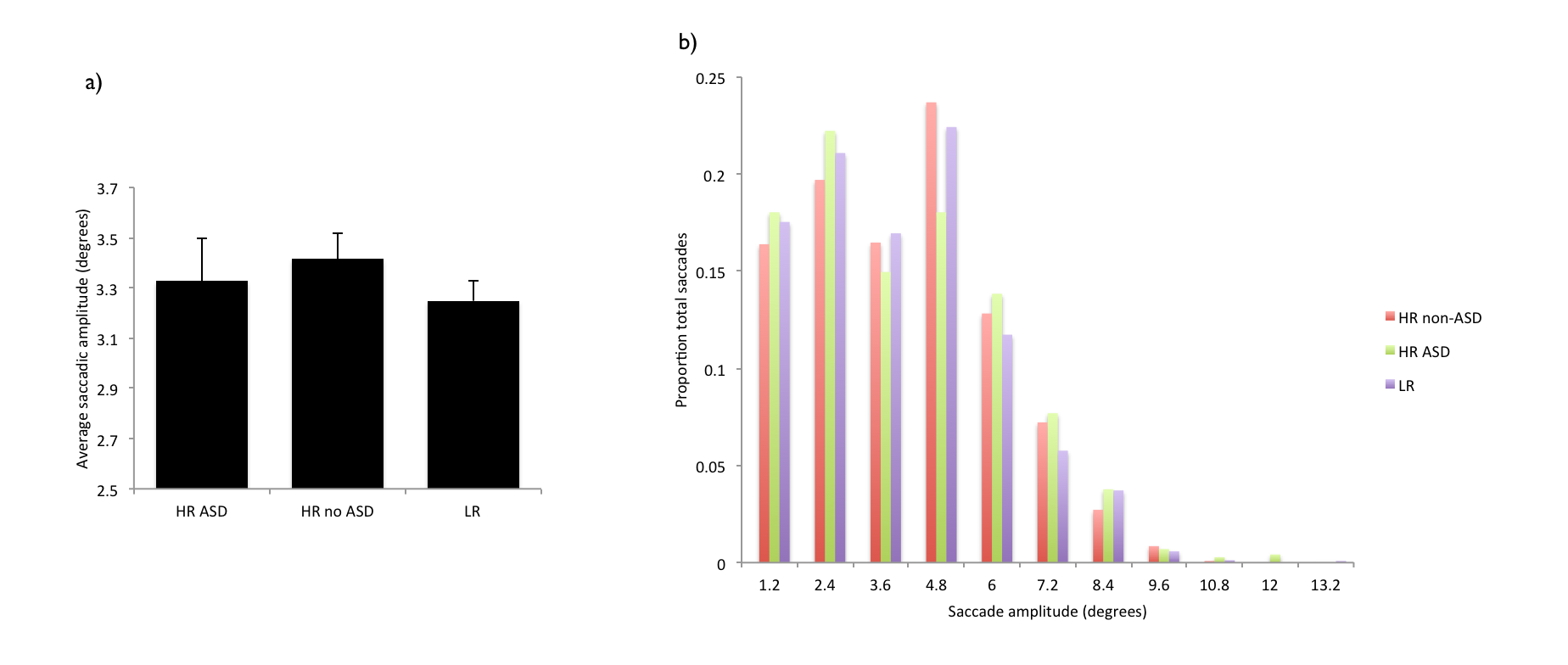


*Figure S2. a) Bar chart showing average saccadic amplitude by group. b) frequency distribution showing saccadic amplitude by group. Units are degrees of visual angle.*

*Part 4 - Variability in fixation duration*

*a) Ex-Gaussian distribution fitting*. Figure S3a shows the frequency distributions of the fixation durations obtained. Ex-Gaussian distributions were fitted to the per-group fixation duration distributions shown in Figure S3a using Matlab algorithms from Lacouture & Cousineau (2008). The ex-Gaussian distribution is a widely used technique for quantifying response time distributions (cf. e.g. Dawson, 1988; Geurts et al., 2008; Unsworth et al., 2011). The resulting ex-Gaussian function has three parameters, μ, σ, and τ. The two first parameters (μ and σ) show the mean and standard deviation of the Gaussian component, which approximates to mode or variance of the response time distribution. The third parameter (τ) is the exponential component, which approximates to the ‘tail’ of the distribution (Lacoutre & Couineau, 2008). The parameters obtained for HR/LR were μ =0.16/0.16, σ =0.024/0.023, τ =0.34/0.39, suggesting that the difference between the distributions may be relatively more driven by differences in the tau component of the distribution, approximating to the skewedness, or tail. However, these fittings could not be performed on a participant-by-participant basis due to the relatively small number of fixation durations obtained per individual, which exploratory analyses suggested rendered distribution fitting unreliable.


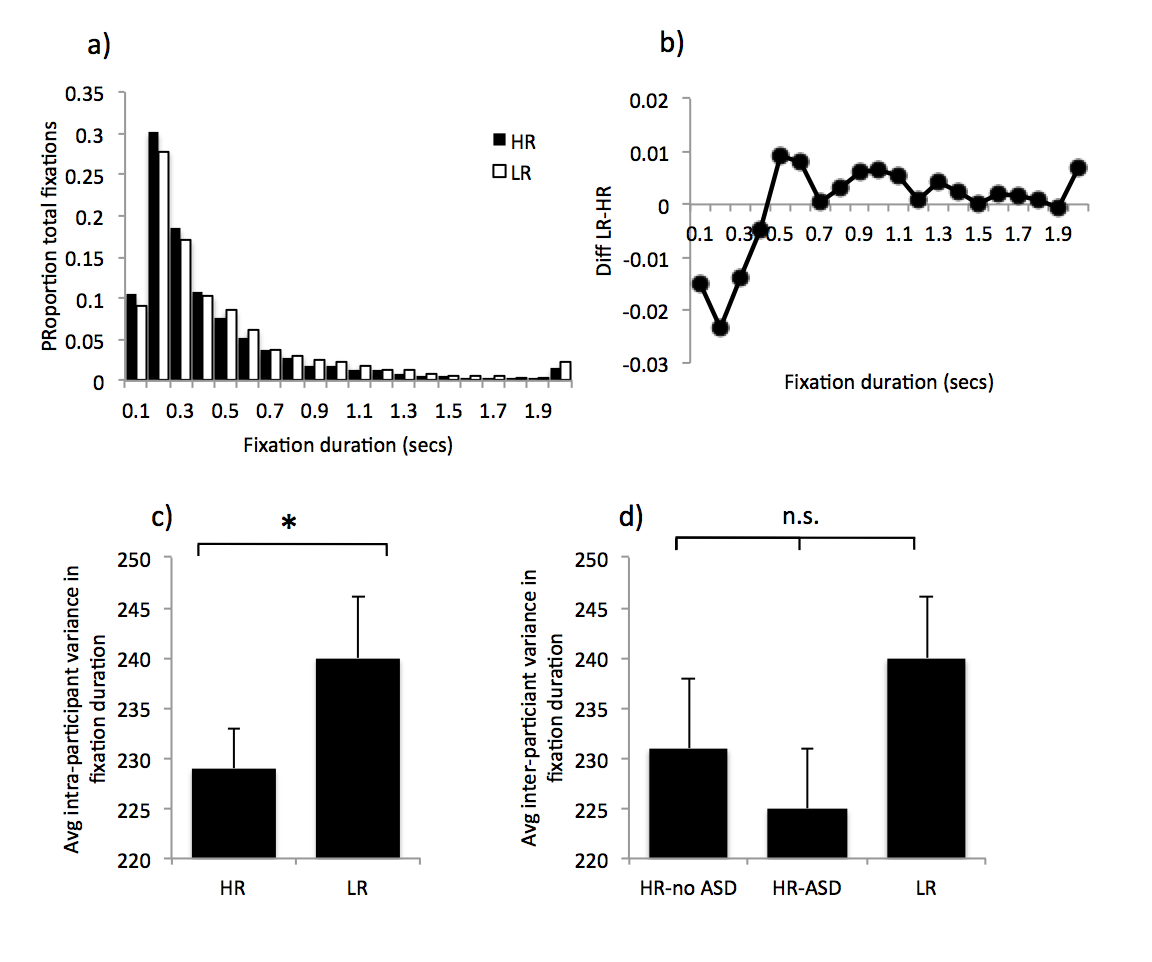


Figure S3. a) Frequency distribution of all fixation duration data, divided by risk group. These distributions show all valid fixations obtained, pooled by group. They do not show the single per-participant medians on which our main statistical analyses were conducted (these are shown in Figure S1). b) The difference between the frequency distributions for the HR and LR infants shown in Figure a. c) Average intra-individual variance in fixation duration. Results have been weighted by number of fixations obtained as described in the text. d) Average intra-individual variance in fixation duration by outcome group (just Cohort 1).

*b) Intra-individual variance in fixation duration*. The differences observed above could be caused either by increased intra-individual or by increased inter-individual variation in the LR group. In order to distinguish between these possibilities we calculated intra-individual variance in fixation duration (see Figure S3). A univariate ANOVA was performed separated by risk/outcome group and Cohort (1,2). Since intra-individual variance measures are heavily influenced by a small number of outliers, fixations greater than 1200ms were excluded from this analysis (following Nuthmann et al., 2010). A bivariate ANOVA on fixation duration weighted by number of fixations by Risk (HR, LR) and Cohort (1,2) revealed that the HR group showed significantly lower intra-individual variability in fixation duration (*F*(1,185)=7.51, *p*=0.007, η^2^=.039) which did not differ by Cohort (main effect of Cohort: *F*(1,185)=.001, *p*=0.98; interaction Cohort x Risk: *F*(1,185)=.18, *p*=.68). A second univariate ANOVA on fixation duration weighted by number of fixations by Outcome (HR- ASD, HR-no ASD, LR) showed a significant effect of Outcome (*F*(1,91)=3.69, *p*=0.029, η^2^=.075). However, posthoc tests indicated that this was driven by HR vs LR group differences rather than by differences within the low risk group (HR- no ASD vs LR, p=0.06; HR-ASD vs LR p = 0.1; HR-no ASD vs HR ASD p = 0.99; see Figure S3d).

*c) Variance in fixation duration – change in fixation duration over time*. Following previous research (Unema et al. 2005; Pannasch et al., 2008; Tatler & Vincent, 2008) we wished to calculate the change in the fixation duration between the first and second half of each trial. Two exclusions were necessary for this analysis (see Figures 3b and 3c). First, experimenter error led to inaccurate recordings of the start times of trials for a subset of participants, meaning that they were unavailable for this analysis (24 HR, 2 LR). In order to assess whether the mean fixation durations of those participants excluded from this analysis differed from those participants included, a bivariate ANOVA was conducted on fixation duration weighted by number of fixations, with Risk (HR, LR), Cohort (1,2) and Exclusion (included or excluded from the analysis of change over time) as the independent variables. There was no significant main effect of Exclusion (F(1,181)=.2.99, p = 0.085, η^2^=.016 ). This suggests that there were no systematic differences in fixation duration between children included versus excluded from analyses of change over time.

Second, those infants who did not provide more than 12 valid fixations per half were excluded, since weighting of the type described above was impractical for difference score analyses. Table 1 and Figure S1 presents a cohort-by-cohort breakdown of our results, showing that differences were observed consistently across Cohort 1 and 2; data from Cohorts 1 and 2 were included in the same ANOVA to increase power due to the lower sample size. A bivariate ANOVA on the difference in fixation duration between the first and second halves of the trial by Risk (HR, LR) and Cohort (1,2) found a significant main effect of Risk (*F*(1,113)=3.96, *p*=.049, η^2^=0.034). This indicates that the LR group showed a greater increase in fixation duration across the two halves of each trial than the HR group. There was no main effect of Cohort (*F*(1,113)=.21, *p*=.65); and the interaction of Cohort by Risk was not significant (*F*(1,113)=0.002, *p*=.97). We subsequently examined the difference in fixation duration between the first and second halves of the trial for each risk group separately. One-way ANOVAs by Cohort (1,2) indicated that the LR group showed longer fixation durations in the second compared to the first half of each trial (*F*(1,47)=4.18, *p*=0.047, η^2^=0.08), but the HR group did not (*F*(1,66)=0.21, *p*=0.65, η^2^=0.001) (see Figures 3b and 3c). An identical analysis was conducted to look at outcome group effects (HR-ASD vs HR-noASD vs LR) in Cohort 1; this did not reach significance (*F*(1,57)=1.21, *p*=.30, η^2^= 0.041; see Figure S4).


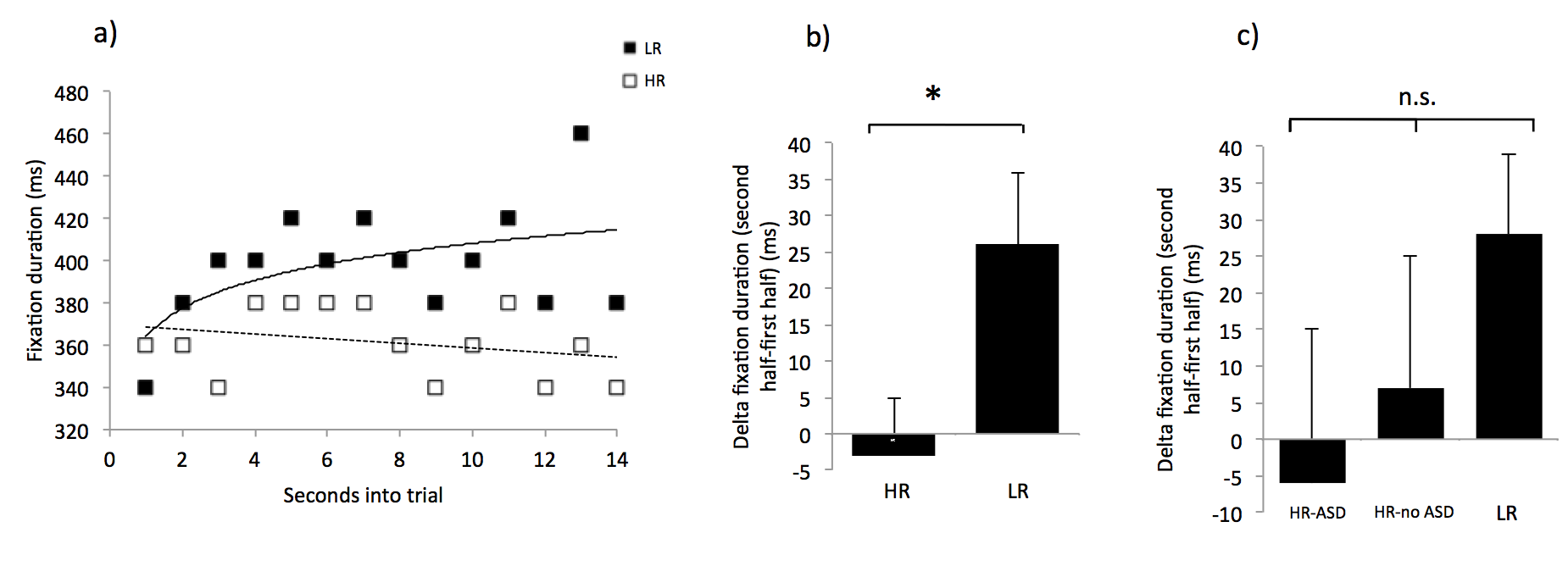


*Figure S4: Static images were presented for 15000ms per trial. a) All fixations have been subdivided according to the second in which the fixation was initiated. Bin 1 shows all fixations that were initiated between 0 and 1000ms into the trial, and so on. Fixations initiating later than 14000ms into the trial have been excluded due to cropping effects. Within each bin the median fixation duration of all the fixations that were collected from HR and LR infants has been recorded. For the LR group the best fit was an exponential curve (F(1,12)=9.42, p=.01), which is similar to the findings reported for neuro-typical adults by Unema et al. (2005); for HR infants, in contrast, the exponential curve was not a good fit to the data (F(1,12)=.002, p=.96); a linear regression line has been plotted. b) The difference in fixation duration between the first half and second half of each trial, pooled across the two Cohorts. The star shows the significance of the ANOVA reported in the main text (p<0.05). c) The same measure as in Figure 3b, by ASD outcome.*

3) Supplementary References (only given where not listed in the main references)

American Psychiatric Association. (2013). Diagnostic and statistical manual of mental disorders (5th ed., text rev.). Washington, DC: Author.

Dawson, M. R. W. (1988). Fitting the ex-Gaussian equation to reaction-time distributions. *Behavior Research Methods Instruments & Computers* *20*(1): 54-57.

Durand, F. and J. Dorsey (2002). Fast bilateral filtering for the display of high-dynamic-range images. *ACM Transactions on Graphics* 21(3): 257-266.

Geurts, H. M., Grasman, R. P. P. P., Verte, S., Oosterlaan, J., Roeyers, H., van Karnmen, S. M., et al. (2008). Intra-individual variability in ADHD, autism spectrum disorders and Tourette's syndrome. *Neuropsychologia*, *46*(13), 3030-3041.

Goodman, R., T. Ford, et al. (2000). The Development and Well-Being Assessment: Description and initial validation of an integrated assessment of child and adolescent psychopathology. *Journal of Child Psychology and Psychiatry and Allied Disciplines* *41*(5): 645-655.

Griffith, E. M., B. F. Pennington, et al. (1999). Executive functions in young children with autism. *Child Development* *70*(4): 817-832.

Lacoutre, Y. & Cousineau, D. (2008). How to use MATLAB to fit the ex-Gaussian and other probability functions to a distribution of response times. *Tutorials in Quantitative Methods for Psychology*, *4*(1), 35-45.

Rodriguez Saez de Urabain, I., Johnson, M. H. & Smith, T. J. (2014) GraFIX: A semi-automatic approach for parsing low and high quality eye-tracking data.. *Behavior Research Methods, DOI 10.3758/s13428-014-0456-0*

Rutter, M. (2003). Social Communication Questionnaire. (WPS: Los Angeles).

Unsworth, N., Redick, T. S., Lakey, C. E., & Young, D. L. (2011). Lapses in sustained attention and their relation to executive control and fluid abilities: An individual differences investigation. *Intelligence*, 38(1), 111-122.

World Health Organization (1993). The ICD-10 classification of mental and behavioural disorders: diagnostic criteria for research.
